# PDMAA Hydrogel Coated U-Bend Humidity Sensor Suited for Mass-Production

**DOI:** 10.3390/s17030517

**Published:** 2017-03-04

**Authors:** Christian Kelb, Martin Körner, Oswald Prucker, Jürgen Rühe, Eduard Reithmeier, Bernhard Roth

**Affiliations:** 1 Hannover Centre for Optical Technologies, Leibniz University Hannover, Nienburger Straße 17, 30167 Hannover, Germany; sekretariat@hot.uni-hannover.de (E.R.); bernhard.roth@hot.uni-hannover.de (B.R.); 2 Institut für Mikrosystemtechnik - IMTEK, Chemistry and Physics of Interfaces, University of Freiburg, Georges-Köhler-Allee 103, 79110 Freiburg, Germany; martin.koerner@imtek.uni-freiburg.de (M.K.); prucker@imtek.de (O.P.); ruehe@imtek.uni-freiburg.de (J.R.); 3 Institute for Measurement and Automatic Control, Leibniz University Hannover, Nienburger Straße 17, 30167 Hannover, Germany

**Keywords:** waveguides, polymer optics, planar systems, humidity sensor, evanescent wave sensor

## Abstract

We present a full-polymer respiratory monitoring device suited for application in environments with strong magnetic fields (e.g., during an MRI measurement). The sensor is based on the well-known evanescent field method and consists of a 1 mm plastic optical fiber with a bent region where the cladding is removed and the fiber is coated with poly-dimethylacrylamide (PDMAA). The combination of materials allows for a mass-production of the device by spray-coating and enables integration in disposable medical devices like oxygen masks, which we demonstrate here. We also present results of the application of an autocorrelation-based algorithm for respiratory frequency determination that is relevant for real applications of the device.

## 1. Introduction

The utilization of polymer-based optical systems for the measurement of different mechanical [1] or environmental [2,3] parameters has been and still is an intensively researched subject over the past ten years. Although the achievable precision is usually lower than with glass optical devices, polymer-only systems excel in cost-efficiency, mechanical flexibility, and variety of possible shapes. Especially due to the desired compatibility between utilized materials and production processes such as hot-embossing [4] or roll-to-roll applications [5], the search for easily-processable light-guiding substrate and sensing materials is still ongoing.

Due to their chemical inertness and their insensitivity towards strong electric or magnetic fields, optical systems for humidity measurement are well-suited to monitoring tasks in harsh environments such as in the vicinity of or inside a magnetic resonance tomography device (MRI). Here patients often receive depressants to attenuate the experience with the often very loud devices and long measurement times. Overdosage of these drugs could lead to respiratory depression [6]—a condition that is especially dangerous when the view onto the patient is obstructed, such as during an MRI measurement.

Optical humidity sensors based on evanescent field sensing have been demonstrated with a multitude of different material combinations. As early as 1996, Gupta et al. [7] presented a U-bend refractive index sensor based on unclad silica multimode fiber. This measurement principle can be found in U-bend humidity sensors that measure the varying refractive indices of water-absorbing cladding materials like agarose gel, porous solgel silica, or CoCl_2_-doped polymer [8,9,10]. Some demonstrated measurement principles replace the U-bend with a tapered region and utilize either single-mode silica fiber [9] or multi-mode silica fiber [8,10]. Multiplexing of this type of humidity sensor was demonstrated using additional devices, such as multi-mode arrayed waveguide gratings [11]. A comprehensive review of the demonstrated fiber-optical humidity sensors was given by [12].

Here, we demonstrate a U-bend humidity sensor consisting of a 1 mm plastic optical fiber (POF) clad with a new type of sensory material in the sensing region. The cladding material is a hydrogel coating based on poly-dimethylacrylamide (PDMAA). This material is first deposited by dip-coating on a substrate and then crosslinked in the second step via UV irradiation, which triggers a C,H insertion reaction between UV active benzophenone (MABP) groups incorporated in the polymer with adjacent chains. At the same time, an anchoring of the layer to the polymeric substrate is achieved through the same reaction.

Our chosen approach results in a polymer-only system for relative humidity measurement that we demonstrated originally in [2]. Here, we demonstrate the feasibility of the proposed device as a sensor for respiratory monitoring, included in a medical oxygen mask. The absence of metallic or electronic parts allows the application of the sensor near or inside an MRI device, while the polymer-only approach opens the path for mass-production using a combination of either lamination or hot-embossing and spray-coating techniques, as shown in [13].

## 2. Humidity Measurement with Evanescent Wave Sensors

The underlying principle of evanescent field humidity measurement is the detection of the refractive index variation of a detector waveguide’s core and cladding materials. With a given core refractive index of *n*_1_ = 1.49 (in our case) and a numerical aperture of NA = 0.58 [14] of the fiber utilized for connection to and from the sensing region (an ESKA BH-4001 POF), the cladding refractive index can be determined to be $n_{2}=1.37$ and the critical angle of total internal reflection (TIR) to be $\theta_{c}=66.8{}^{\circ}$. Since a poly(methyl methacrylate) (PMMA) monofilament is utilized in the sensing region, with a surrounding air refractive index of $n_{2}=1.00$, the new critical angle would then be $\theta_{c}=42.1{}^{\circ}$, and therefore not all modes that could possibly be guided are thus excited in the actual sensing region. Since the sensitivity of evanescent field sensors relies on the penetration depth of the evanescent field outside the core, it is desirable to increase the penetration depth $d_{p}$ by decreasing the contact angle, as deduced in [12] and given by the equation

(1) dp=λ2πn1[sin2θ−(n2/n1)2]12

**Figure 1:**
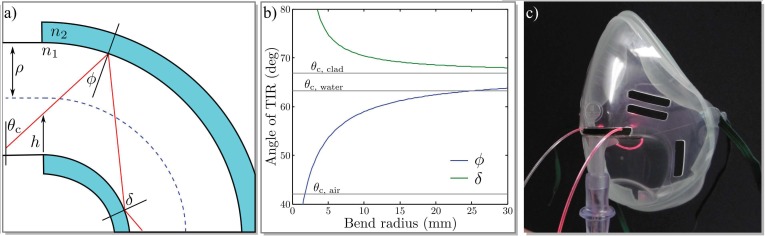
(**a**) Path of light inside the U-bend; (**b**) Angle of total internal reflection (TIR) over bend radius; (**c**) Photograph of the modified oxygen mask fitted with the U-bend humidity sensor.

This can be achieved by applying a U-bend to the monofilament, as depicted in Figure 1a. Since a comprehensive investigation of the geometrical characteristics is given in [7,15], we will restrict our description here to a brief summary. Depending on the entrance height h and the beam angle θ that the individual beam exhibits upon entrance to the bent region with respect to the surface normal, the new TIR angle is

(2) $$\phi =\sin^{-1}\left(\frac{R+h}{R+2\rho }\sin\theta \right)$$

in the case of the outer boundary and

(3) $$\delta =\sin^{-1}\left(\frac{R+h}{R}\sin\theta \right)$$

in the case of TIR on the inner boundary. Note that the description is simplified to a two-dimensional waveguide, thus ignoring skew rays. Figure 1b shows the angles of TIR for the outer and inner boundary over the bend radius of the waveguide, simulated for h=ρ/2 and ρ=0.5 mm.

Plotted in gray are the critical angles for the connecting ESKA Waveguide, $\theta_{c,\;\mathrm{clad}}=66.8{}^{\circ}$, for the monofilament in air, $\theta_{c,\;\mathrm{air}}=42.1{}^{\circ}$, and for the monofilament in water with n=1.33, $\theta_{c,\;\mathrm{water}}=63.2{}^{\circ}$. It can be seen that for a bend radius as large as R=25 mm, the angle of TIR on the outer boundary, ϕ, falls below the critical angle for the monofilament in water, and the beam will not be guided anymore. Thus, the highest possible modes are excited and the penetration depth of the evanescent field is maximized.

Since we do not know the water content of our cladding material exactly, and because the refractive index of the dry cladding material is dependent on the crosslinker content and degree of crosslinking, it is difficult to predict the exact critical angle in the sensing region. However, we can safely assume that the refractive index of the cladding material will not be lower than that of pure water. This leads to the assumption that a bend radius somewhere below R=25 mm is sufficient to excite all higher modes in the monofilament, so that the sensitivity towards refractive index changes in the cladding material is maximized.

Furthermore, a much smaller bend radius would shorten the path length where the PDMAA cladding is applied, and thus decrease the interaction length of higher modes and the sensitive cladding, which in turn would lead to decreased sensitivity. In contrast to this and from a mechanical point of view, a smaller bend radius results in sensors that are mechanically more stable. Since unwanted deformation of the sensor would undoubtedly influence the measurement, we settled for two bend radii of R=10 mm and R=5 mm, which is a compromise between mechanical stability and optical sensitivity.

## 3. Experimental Setup

**Figure 2:**
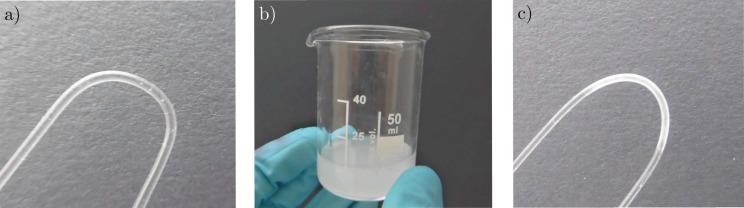
(**a**) The PMMA monofilament after being bent using a heat gun; (**b**) The poly-dimethylacrylamide (PDMAA)–ethanol solution; and (**c**) the coated monofilament after UV-curing.

The U-bend was fabricated using a 1 mm PMMA monofilament with a refractive index of 1.489 @ 632 nm, which was bent to a radius of 5 mm using an aluminum cylinder of appropriate radius and a heat gun. In a second step, the finished bend was coated with PDMAA by dip-coating it into a PDMAA–ethanol solution consisting of 20 mg PDMAA for each mL of ethanol. We let the ethanol evaporate at room temperature and cured the cladding using a 245 nm UV light source over several minutes, ensuring the highest possible degree of crosslinking. Figure 2 shows the different steps from left to right. To evaluate the influence of the bend radius, we also produced a second sensor with 10 mm bend radius in the same fashion. In essence, this demonstrates that the production technology can be greatly simplified to be very resource- and time-efficient, as required for future low-cost and high-throughput applications.

**Figure 3:**
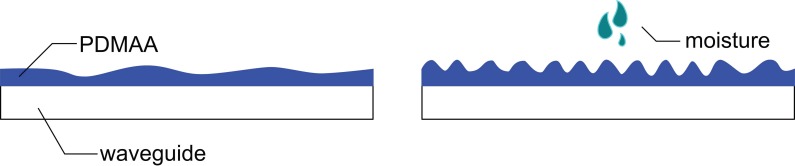
Behavior of the PDMAA cladding material upon water absorption. Due to an increase in volume, the surface roughness increases, leading to increased light scattering, which can either be detected as intensity loss of the propagating light field or as increase of the light signal in the environment of the U-bend.

Apart from the change in refractive index, as discussed in Section 2, the influence of ambient humidity on the PDMAA coating—and thus on the transmitted light—could be determined or at least in part influenced by volumetric swelling of the cladding material. As depicted in Figure 3, a volume increase due to the absorption of water would lead to increased surface roughness of the cladding. For increasing water content, the cladding would then scatter more light, coupled into it by the evanescent field, causing an intensity drop in transmitted light at the output of the sensing region. In [2], we could identify a variation in the scattering behavior upon water absorption.

The change in the refractive index of the cladding material with increasing water content would act contrary to this effect. For an increasing weight component of water, the refractive index of the cladding will decrease, starting at approximately *n* = 1.516 in the dry state with a lower limit of 1.33, which is the refractive index of water [16,17]. This would lead to a lower critical angle in the sensing region, which would result in an increase of transmitted power. Note that the dry refractive index (RI) as reported by [16] is higher than that of the PMMA bare fiber. Initial tests conducted outside a respiratory monitoring setup showed no sharp step in transmission of the sensor for an increase of the air’s relative humidity. This would be expected once the cladding RI drops below the core RI. We therefore suspect the cladding RI to be permanently below 1.49. Since direct measurement of the RI proves difficult due to the high scattering and unclear water content of the coating, we plan to investigate this topic more thoroughly in further work, focusing here on the feasibility of the proposed system as a respiratory monitoring device.

By carrying out initial tests, we found that the sensors built for this work show increasing transmittance with increasing humidity, indicating the decline in cladding refractive index as the main contributor to modulation of the transmitted light.

We prepared a medical oxygen mask with an in-house 3D-printed attachment that holds our prefabricated PMMA U-bend (see Figure 2), positioning its bent region between mouth and nostrils of the mask’s wearer to ensure monitoring of both breathing through the nose or the mouth. Incoherent light from a Thorlabs^®^ M625F1 High Power LED (Thorlabs Inc, Newton, New Jersey, NJ, USA) is coupled into the sensor, and the transmitted power is monitored by a BPW 34 photodiode (Osram GmbH, Munich, Germany) connected to a PDA 200 C photodiode amplifier (also Thorlabs). All devices prove rather cost-efficient when compared, for example, to the standard interrogator setup utilized for the readout of fiber Bragg grating (FBG) humidity sensors.

## 4. Results and Discussion

**Figure 4:**
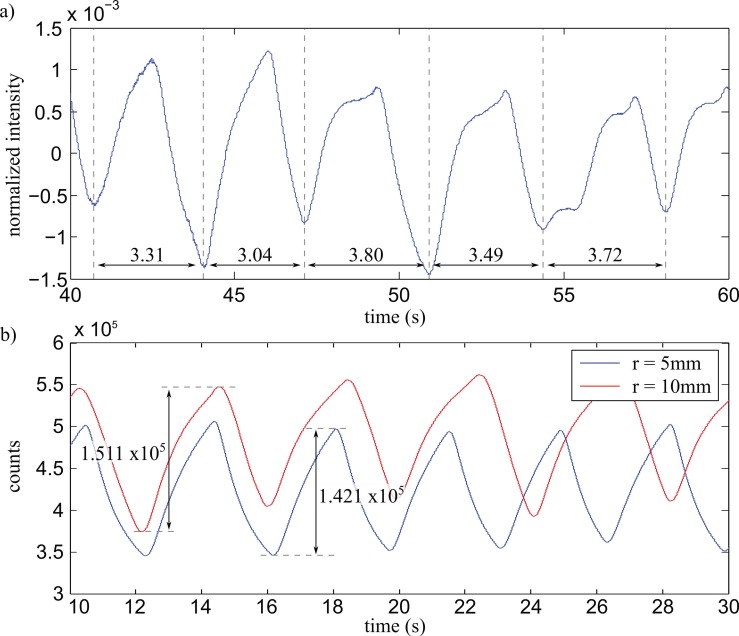
(**a**) Normalized measurement data of a person breathing inside the oxygen mask, filtered with a discrete first-order low pass filter (PT1) filter; (**b**) Absolute measurement data in counts, comparing two different U-bend sensors—one with a radius of 5 mm and one with a radius of 10 mm.

As shown in Figure 4a, the intensity transmitted through the sensor increases with increasing humidity—in this case caused by exhalation of the mask’s wearer. With the person sitting idle, a drop in intensity could be monitored roughly every 3.5 s after applying a first-order low pass filter (PT1) with a time constant of 1 s to the signal. To investigate the stability of the PDMAA cladding, we repeated the measurements with the same sensors after 4 weeks, finding no discernible difference in sensitivity or reaction time. To automate the process of measuring the breathing frequency during the measurement as would be desirable in a clinical environment for monitoring a patient’s breathing frequency (e.g., to detect the onset of panic during an MRI measurement), we utilized a new read-out approach initially developed in the field of speech signal processing [18].

Figure 4b shows a comparison between two sensors with bend radii of 5 mm and 10 mm. As can be observed, there does not appear to be a significant increase in sensitivity between the two sensors. For multi-mode waveguides as utilized here, this result can be explained with all possible modes (i.e., reflection angles) already fully occupied with equal intensity. A decrease of the bend radius would cause more light to couple out in the sensing region, not contributing to the measurement, while all possible reflection angles up to the critical angle are still equally occupied and the energy in the evanescent field stays the same.

With the irregular and very low breathing frequency, the standard Fourier-transform method would need several tens of seconds to reliably calculate the fundamental breathing frequency, resulting in a poor dynamic range of the measurement system. Looking into the field of speech signal processing, Staudacher et al. proposed a method to quickly determine the fundamental frequency of a speech signal using an adaptive autocorrelation approach [18]. This approach is meant to quickly identify the pitch of a speech signal (e.g., to detect questions, which normally end with a rise in pitch towards the end of the sentence) in a real-time speech processing application.

The functioning principle of the utilized approach is based on a segment-wise autocorrelation of the real-time signal. A segment of the measured signal of a certain length is stored and the correlation with the live signal is computed until a maximum is detected. The in-time difference between the current maximum and the last indicates the breathing rate. The length of the stored signal is inversely proportional to the lowest detectable frequency component.

**Figure 5:**
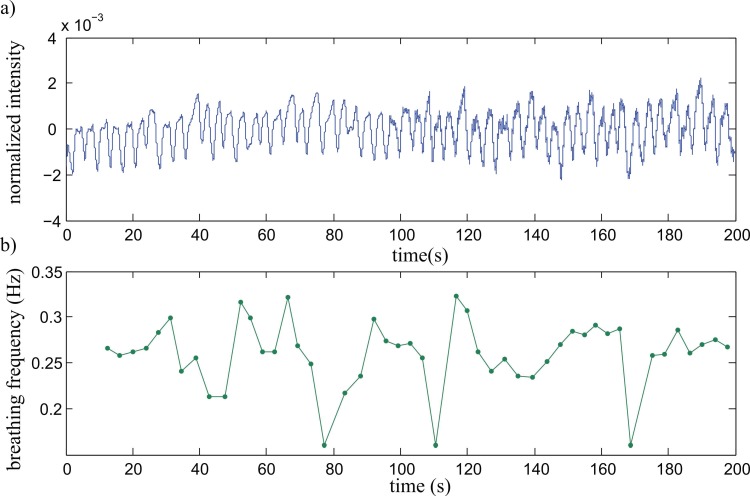
(**a**) Measurement data over 200 s showing regular breathing and (**b**) fast adaptive evaluation of the breathing frequency using an audio processing approach [18].

Figure 5a shows a signal with a length of 200 s of the sensor’s intensity output, measured with the person sitting idle, breathing at will through the nose or the mouth. Although the signal is irregular, there is no clear distinction possible between mouth or nose breathing, proving the feasibility of the design for monitoring both breathing cases. Figure 5b shows the detected breathing frequency with an average of ~0.25 Hz with a maximum of 0.32 Hz and a minimum of 0.16 Hz.

To test the sensor’s response to longer periods of suspended respiration and increased CO_2_ content of the air it is in contact with, we carried out experiments where the patients held their breath for a few tens of seconds and carried on with normal breathing afterwards.

**Figure 6:**
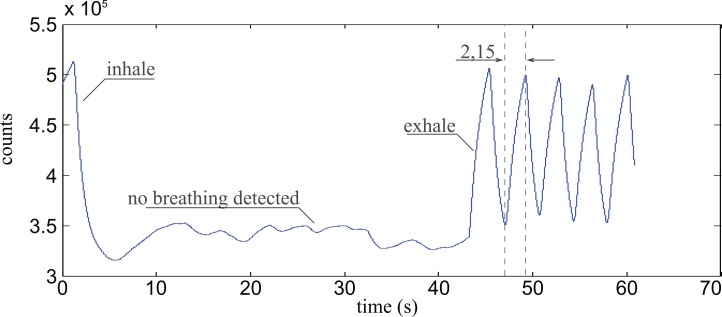
Sensor signal for a sensor with 5 mm bend radius showing a patient holding their breath for approximately 40 s. The autocorrelation algorithm does not detect a breathing frequency during the time the breath is held.

As soon as the patient’s breathing ceases, the sensor shows a response resembling a random walk at slightly increased intensity levels, indicating slightly increased humidity, most likely originating from the patient’s skin and nasal mucosae, as can be observed in Figure 6. Additionally, from the time between valley and peak a rise time of 2.15 s can be calculated, which is a satisfactory value for humidity sensors.

Note that we found drops of condensed water inside the mask after a series of tests. In the event that these drops would accumulate on the sensor itself, a decrease in signal strength would occur. Since the utilized algorithm can extract self-similarities from the signal segments even for low amplitudes, the measurement would not be affected for a while—with further decreasing amplitudes, the sensor would report cease of respiration, thus generating a false positive.

### Temperature and Water Sensitivity

Since non-functionalized step-index multi-mode fibers were utilized as intrinsic temperature sensors in a couple of works [15,19], a combined sensitivity of our sensor towards relative humidity and temperature is possible. In both works, a U-bend is applied to the fiber to lower the local numerical aperture and thereby increase the sensitivity towards temperature changes. In one work, the different prefixes of the thermo-optical coefficients of a silica glass fiber and its silicone rubber cladding is utilized for temperature detection [19]. With the smaller differences between the thermo-optical coefficients of the core and the fluorinated cladding of standard-POF, temperature detection is also possible [15]. However, in the second case, a rather small radius of the U-bend of around 1 mm is necessary, and the readout is done by comparing the intensity variations of two different transmitted wavelengths which are utilized to increase sensitivity. With the comparatively small temperature differences between room- and body-temperature, our device is confronted with an additional attenuation effect stemming from temperature variations which can be neglected. However, it is worth mentioning that for the precise measurement of humidity, the effect should again be taken into account and studied in detail.

Besides temperature, the absorption of environmental water could also influence the measurement of the respiratory monitoring device. This could hold true especially for PMMA materials where water absorption in PMMA can be as high as 3.5%. As early as 1985, an investigation was carried out on absorption losses in polymer optical fiber [20], resulting in an increase in absorption losses of 15 dB/km at wavelengths between 614 nm and 668 nm for an increase in relative humidity from 30% RH to 90% RH. Converted to the path length of the coated length of our sensor (15.7 mm and 31.4 mm for bend radii of 5 mm and 10 mm, respectively), we obtain additional absorption of $2.36\;\times \;10^{-4\;}\mathrm{dB}/\mathrm{mm}$ and $4.71\;\times \;10^{-4}\;\mathrm{dB}/\mathrm{mm}$.

From Figure 4b, a peak-to-peak difference of 36% relative to the signal maximum can be observed. This amounts to an attenuation change of −5.6 dB. Thus, additional attenuation caused by water absorption into the monofilament material is an effect that does not influence the performance of the proposed design.

## 5. Outlook and Conclusions

In this work, we demonstrated a prototype optical breathing sensor based on a U-bend humidity sensor which was placed inside an oxygen mask. With the described setup, we were able to monitor a person’s breathing activity regardless of oral or nasal breathing or a combination thereof. Furthermore, we successfully applied a fast autocorrelation algorithm to the sensor’s intensity signals, demonstrating fast detection of breathing frequency within one breathing period.

While the measurements for this prototype were carried out with a 1 mm POF which is rather bulky, we ultimately aim for a fully planar humidity sensor compatible with hot embossing or lamination production technologies in the future. We recently showed that it is possible to fabricate polymer-optical waveguides by utilizing a lamination process, a cyclic olefin polymer (COP) filament as core, and a PMMA foil as cladding material. Since the process relies solely on spray coating and lamination technologies, it is well suited for larger-scale production of the demonstrated humidity sensor using roll-to-roll compatible technology. In more detail, the sensing polymer can be applied by spray coating, and the resulting sensing waveguide can then be laminated between two PMMA substrate foils—one with an opening allowing to establish contact between the coated filament and the surrounding for later humidity measurement.

**Figure 7:**
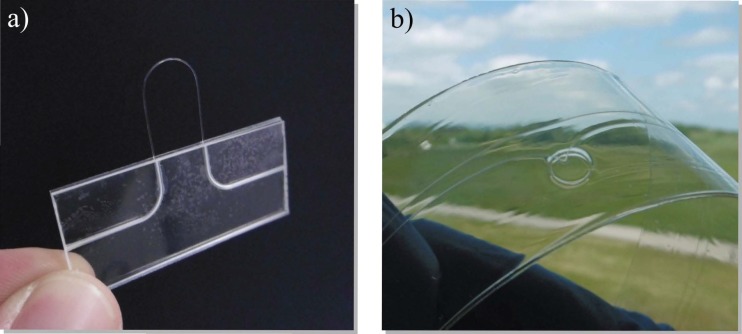
(**a**) Photograph of a U-bend humidity sensor utilizing a 250 µm core plastic optical fiber (POF) with stripped cladding in the sensing region and (**b**) a laminated device with 100 µm cyclic olefin polymer bare fiber laminated between two PMMA substrate layers.

Figure 7 shows two possible manifestations of the U-bend sensor presented here. On the left, a 250 µm POF with stripped cladding in the sensing region is embedded between two PMMA substrates, structured by hot embossing before assembling. The whole device can be placed on a circuit board between a side-emitting LED and a side-receiving photodiode and thus provide an alternative to classical MEMS devices for humidity sensing. A more flexible laminated device is shown on the right, consisting of a 100 µm cyclic olefin polymer bare fiber embedded between two PMMA substrates via roll-to-roll lamination. A hole in one substrate, situated at the place where the filament is bent, allows environmental humidity to reach the measurement region.

In the next step, we plan to produce the proposed sensors both by lamination and by hot embossing in order to establish the process chain and evaluate and compare the performance of both implementations. In a second step, we will integrate the humidity sensor together with polymer-optical strain sensors on a single substrate to allow for humidity-compensated optical strain measurement; i.e., the realization of more sensitive and reliable polymer-based sensor devices qualified for applications in medicine or production monitoring.
